# Survey of Clinical Practice in Chronic Myeloproliferative Neoplasms in Croatia: A Study by the MPN Working Group Party of the Croatian Cooperative Group for Hematologic Diseases (KROHEM)

**DOI:** 10.3390/jcm14051524

**Published:** 2025-02-24

**Authors:** Ivan Krecak, Marko Lucijanic, Rajko Kusec

**Affiliations:** 1 Department of Internal Medicine, General Hospital of Sibenik-Knin County, 22000 Sibenik, Croatia; 2 Faculty of Medicine, University of Rijeka, 51000 Rijeka, Croatia; 3 University of Applied Sciences, 22000 Sibenik, Croatia; 4 Division of Hematology, University Hospital Dubrava, 10000 Zagreb, Croatia; 5 Department of Scientific Research and Translational Medicine, University Hospital Dubrava, 10000 Zagreb, Croatia; 6 School of Medicine, University of Zagreb, 10000 Zagreb, Croatia

**Keywords:** myeloproliferative neoplasms, Croatia, survey, real-world practice

## Abstract

**Background/Objectives**: Chronic myeloproliferative neoplasms (MPNs), essential thrombocythemia (ET), polycythemia vera (PV), and myelofibrosis (MF), are hematopoietic stem cell disorders characterized by increased proliferation of mature myeloid cells, a chronic inflammatory state, and high cardiovascular risk. The diagnostic and therapeutic landscape in the field of MPNs is rapidly evolving. Therefore, it is important to assess the behavior of physicians involved in the management of MPN patients to gain insight into how they embrace the current diagnostic and treatment landscape in real-life settings. **Methods**: An online anonymous survey consisting of 50 questions regarding their MPN practice and divided into four major domains (physician characteristics, diagnostic procedures, therapeutic decisions, and patient follow-up) was sent through the Croatian Cooperative Group for Hematologic Diseases’ (KROHEM’s) e-mailing list to all Croatian hematologists. **Results**: Thirty-one out of ninety adult hematologists (34.4% response rate) from KROHEM responded to this survey. There was a very high rate of self-proclaimed abidance to current international diagnostic and therapeutic recommendations, with no major differences among academic and community practices. However, some areas of uncertainty have been highlighted, especially in the frequency of cytogenetic and molecular testing, as well as very low implementation of the Myeloproliferative Neoplasm Symptom Assessment Form (MPN-SAF) questionnaire in everyday practice. **Conclusions**: This study provides an important snapshot of the current MPN practice in Croatia. Similar studies from other countries are needed to provide a more detailed overview of real-life MPN practice globally.

## 1. Introduction

Myeloproliferative neoplasms (MPNs) are hematopoietic stem cell disorders encompassing chronic myeloid leukemia (*BCR::ABL1* mutation positive), polycythemia vera (PV), essential thrombocythemia (ET), myelofibrosis (MF; both primary-PMF and secondary, post-PV or post-PV, MF), chronic neutrophilic leukemia, chronic eosinophilic leukemia, juvenile myelomonocytic leukemia, and MPN, not otherwise specified. *BCR::ABL1*-negative MPNs, ET, PV, and MF, are MPN entities with the highest prevalence and are clonally driven by acquired mutations in the Janus kinase 2 (*JAK2*), calreticulin (*CALR*), or myeloproliferative leukemia virus (*MPL*) genes. These disorders are characterized by increased proliferation of mature myeloid cells, frequent splenomegaly, variable degrees of bone marrow fibrosis, debilitating inflammation-linked symptoms, and an increased cardiovascular risk [1,2,3]. Mainly due to adverse cardiovascular events and the propensity to transform to acute myeloid leukemia (AML), life expectancy in all three MPN entities is shorter than that in the general population, especially in MF [4,5]; however, with optimal medical care, many MPN patients may still experience a normal lifespan [6]. Therefore, the main treatment goals in MPNs are mitigating thrombotic risk, symptom control, and preventing disease progression to secondary MF and AML.

Therapeutically, all PV patients receive aspirin and are phlebotomized with an aim to maintain the hematocrit of less than 45% in order to mitigate thrombotic risk; high-risk patients (those older than 60 years of age or with prior thrombosis), as well as low-risk PV patients with persistent or progressive leukocytosis, extreme thrombocytosis, symptomatic disease, and inadequate hematocrit control despite phlebotomies, receive cytoreduction, usually with interferons (IFN) or hydroxyurea (HU). In patients who are refractory or intolerant to HU, the use of ruxolitinib, a *JAK1/JAK2* inhibitor, or IFN (if HU was used in first-line) are recommended [1,7,8]. Patients with ET are usually risk-stratified according to the Revised International Prognostic Score for Thrombosis in ET (R-IPSET) [9] which accounts for age, presence of *JAK2* mutation, and thrombosis history; high-risk patients (those with prior thrombosis or older than 60 years of age and with *JAK2* mutation) receive IFN or HU. Low- to high-risk ET patients also receive aspirin. In the second-line treatment, switching to anagrelide or IFN (in case HU was used in the first-line) is advised. Patients with ET having extreme thrombocytosis (platelet count > 1000–1500 × 10^9^/L) and secondary von Willebrand’s disease may be at higher risk of bleeding and thus also warrant cytoreduction [2,7]. In MF, the most aggressive MPN, the primary focus of prognostication is predicting the risk of death. The most commonly used tool is the Dynamic International Prognostic Scoring System (DIPSS), which can be used anytime during the disease course, incorporates easily available parameters (patient age, total leukocyte count, hemoglobin levels, presence of constitutional symptoms, and peripheral blasts) and delineates four risk categories (low-risk, intermediate-1, intermediate-2, and high-risk). Intermediate-2 and high-risk DIPSS patients have the worst survival rate and are usually referred for an allotransplant; patients ineligible for an allotransplant (because of advanced age or comorbidities) are typically offered ruxolitinib, as this compound has been shown to effectively reduce spleen size, mitigate symptoms, and to potentially improve survival in this subset of patients [3,8,10,11,12]. For MF-associated anemia, erythropoietin, steroids, danazol, lenalidomide (in case of chromosome 5q deletion), momelotinib, or luspatercept may be given alone or in combination with ruxolitinib and other compounds [3,7].

The current diagnostic and therapeutic landscape of MPNs is rapidly evolving. In recent years, mutation- and karyotype-enhanced disease prognostication (i.e., Mutation Enhanced International Prognostic Systems for ET and PV-MIPSS-ET/PV, Genetically Inspired Prognostic Scoring System-GIPSS or Mutation and Karyotype Enhanced International Prognostic Scoring System-MIPSS for MF) has significantly improved the risk stratification of MPN patients [13,14,15]; multiple clinical trials and novel compounds for the treatment of MPN patients became available (i.e., ropeginterferon, rusfertide, fedratinib, momelotinib, and pacritinib) and many are being tested in randomized clinical trials (i.e., givinostat, bomedemstat, imetelstat, pelabresib, navtemadlin, navitoclax, etc.) [1,2,3]. Therefore, it is important to assess the behavior of physicians directly involved in the management of MPN patients to gain insights into how they embrace the current diagnostic and treatment landscape in real-life settings. The aim of this study was to investigate the current MPN clinical practice in Croatia.

## 2. Subjects and Methods

### 2.1. Study Design

An online anonymous survey (*Google Forms^®^* application) consisting of 50 questions regarding MPN practice (full set of questions and answers are shown in Supplementary Table S1) was sent through the Croatian Cooperative Group for Hematologic Diseases’ (KROHEM’s) mailing list to all Croatian hematologists in August 2024 and was closed, after two reminders, in November 2024. The survey was divided into four major domains: (1) physician characteristics, (2) diagnostic procedures, (3) therapeutic decisions, (4) patient follow-up. Respondents completed the questionnaire; the results were transferred to Microsoft Excel^®^ spreadsheet and were then analyzed.

### 2.2. Statistics

Descriptive statistics were used for data analysis and presentation. The chi-square test and Fisher’s exact test were used to analyze differences between categorical variables. A *p* value < 0.050 was considered statistically significant for all presented analyses. Statistical calculations and graph designs were performed with MedCalc Statistical Software^®^ (Ostend, Belgium, v. 23.0.5).

## 3. Result

Thirty-one (41.9% females) out of ninety adult hematologists (34.4% response rate) from KROHEM responded to this survey; the majority (71%) of them were practicing their clinical activity in academic centers and most of them (93.3%) had significant (>5 years) of experience in the field of MPNs. Half of the respondents reported that they currently follow 1–20 ET and PV patients and <10 MF patients in their clinical practice.

Regarding diagnostic procedures, two-thirds of respondents concomitantly order *BCR::ABL1* mutation testing in cases of MPN suspicion. In the case of PV suspicion, *JAK2-V617F* mutation, serum erythropoietin levels, bone marrow biopsy, and cytogenetics are performed by 100%, 83.9%, 77.4%, and 51.6% of respondents, respectively. More than half of respondents also perform *exon 12* mutational testing in cases where *V617F* is negative, and more than half of respondents feel that *JAK2* allele burden testing is important for correct MPN diagnosis, assessment of treatment, and disease prognosis. The majority of Croatian hematologists (93.5%) also test for *CALR* and *MPL* mutations if *JAK2-V617F* mutation is negative in cases of ET and MF suspicion; 73.5% of them also perform bone marrow biopsy when ET is suspected.

Concerning disease risk stratification, >80% of respondents employ the European Leukemia Network (ELN) [7] and the R-IPSET scores for thrombotic risk classification for PV and ET patients, respectively. The most preferred tool for MF risk stratification used by Croatian hematologists is DIPSS (64.5%), followed by International Prognostic Systemic Scoring-IPSS (22.6%), DIPSS-Plus (9.7%), and MIPSS (3.2%). Interestingly, even though cytogenetics are not incorporated into routine ELN and R-IPSET ET and PV risk assessments, more than half of respondents additionally perform cytogenetics in ET and PV patients; expectedly, this proportion was even higher in MF patients (76.7%). Testing for detrimental non-driver myeloid mutations (*ASXL1*, *EZH2*, *SRSF2*, *IDH1/IDH2*) in MF is routinely performed by 45.2% of respondents; 86.7% of them consider them important to further refine the risk of young MF patients.

The majority (71%) of respondents consider hematocrit < 45% as a phlebotomy goal for PV patients; 22.6% of them use target hematocrit < 42% for women. Interestingly, the majority of Croatian hematologists (67.7%) also perform phlebotomies in cases of *JAK2*-positive ET and post-PV MF patients having hematocrit > 45%. Aspirin is routinely recommended to 93.5% and 71% of PV and ET patients, respectively. Conversely, antiplatelets are avoided by physicians, mostly in cases of ET patients < 60 years who are *JAK2*-negative and without prior thrombosis (69.2%), who are young (<40 years of age) (50%), and *CALR*-mutated (23.1%). Croatian hematologists also recommend aspirin to 90.3%, 67.7%, 61.3%, and 48.4% of MF patients with cardiovascular risk factors, prior thrombosis, *JAK2*-mutated MF, and post-PV MF, respectively. Twice or thrice daily aspirin is seldomly prescribed by Croatian hematologists (6.5%). Most physicians (93.5%) responded that they use direct oral anticoagulants in MPN patients.

**Figure 1:**
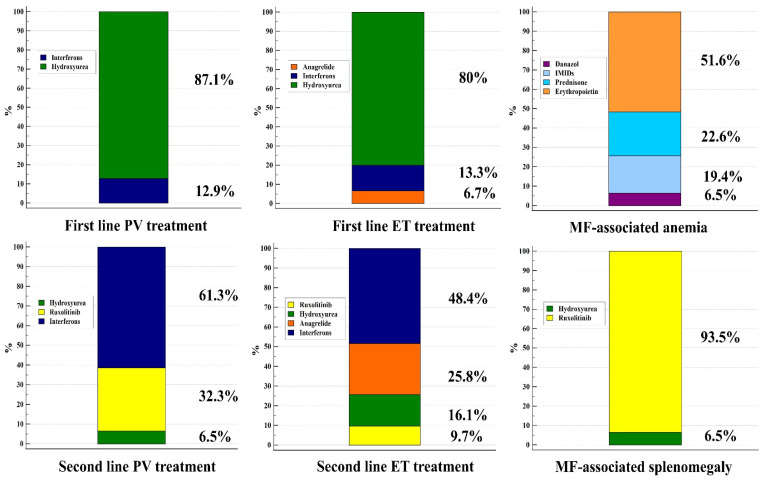
Treatments employed for the first- and second-line treatments in patients with essential thrombocythemia (ET) and polycythemia vera (PV) patients, as well as treatments for myelofibrosis (MF)-associated anemia and splenomegaly.

First- and second-line treatments for MPN patients recommended by Croatian hematologists are shown in Figure 1. As presented, HU is the preferred first-line treatment for 87.1% of PV and 80% of ET patients, whereas in the second-line, IFNs are the most commonly prescribed medications (61.3% PV and 48.4% ET patients). A first-line treatment to manage splenomegaly in MF is ruxolitinib (93.5%), whereas MF-associated anemia is usually treated with erythropoietin (51.6%), followed by prednisone (22.6%), thalidomide/lenalidomide (19.4%), and danazol (6.5%). The most common indication for splenectomy reported by the physicians was painful splenomegaly refractory to cytoreductive treatment and irradiation (96.8%).

Concerning blast phase MPN, most respondents prefer azacitidine/venetoclax (73.3%) for induction treatment, followed “by 3 + 7” (43.3%), and to approximately one-fourth of these patients, Croatian hematologists offer palliation. Two-thirds of respondents enroll MPN patients in clinical trials at their center.

The majority of respondents (>90%) follow low-risk ET and PV patients every 3–6 months; in high-risk disease, the usual follow-up interval shortens to once-monthly in 48.4% and 32.3% of PV and ET patients, respectively. The follow-up intervals for the majority of Croatian hematologists (67.7%) are 1 and 3 months, for intermediate-2/high and low/intermediate-1 risk MF patients, respectively. Most physicians (>90%) repeat bone marrow biopsies only in suspicion of disease transformation; non-driver myeloid mutations are rarely repeated during follow-up (12.9%). To assess spleen size, most respondents use ultrasound (51.6%), followed by palpation (25.8%) and computerized tomography (22.6%). Only 16.1% of respondents routinely use the Myeloproliferative Neoplasm Symptom Assessment Form (MPN-SAF) questionnaire to assess symptom burden in MPN patients.

**Figure 2:**
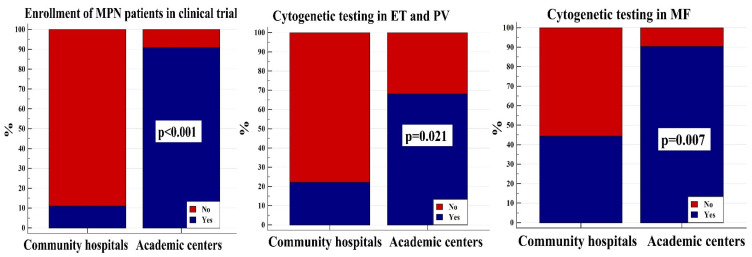
Differences in academic and community center physician-reported enrollment of patients with *BCR::ABL1* negative myeloproliferative neoplasms (MPNs) into clinical trials and diagnostic cytogenetic testing. ET = essential thrombocythemia; PV = polycythemia vera; MF = myelofibrosis.

Finally, physicians in academic centers more often perform cytogenetics in ET and PV (68.2% vs. 22.2%; *p* = 0.021) as well as in MF patients (90.5% vs. 44.4%; *p* = 0.007), and significantly more often enroll patients in clinical trials (90.9% vs. 11.1%; *p* < 0.001), as shown in Figure 2, whereas there were no statistically significant differences in other domains of MPN management (*p* > 0.050 for all analyses). These differences are most probably due to easier access to specialized diagnostic infrastructure in academic centers.

## 4. Discussion

This is the first study to analyze physician preferences regarding MPN management in Croatia and one of the few studies globally [16,17,18,19] which assessed the abidance of physicians to MPN treatment recommendations in real-life settings. Aside from ours, only one study, from Italy [16], comprehensively analyzed all three MPN entities. In our study, we found a very high rate of self-proclaimed abidance to current diagnostic and therapeutic recommendations, with no major differences among academic and community practices. The results presented are quite similar to previously reported surveys regarding real-life MPN practice among Italian [16], German [17], Canadian [18], and United States [19] physicians and demonstrated quite homogeneous MPN management in Croatia, as well as uniform MPN practices globally. Specifically, the vast majority of physicians caring for MPN patients in Croatia follows the World Health Organization criteria for MPN diagnosis [20], proposed risk stratifications [1,2,3,7,9], and treatment recommendations [1,2,3,7,8]. Notably, two-thirds of Croatian hematologists enroll MPN patients in clinical trials, more so in academic centers, probably due to easier access to specialized diagnostic infrastructure. It should also be noted that the majority of clinicians (71%) responding to this survey were from university hospitals, which may also confound the results regarding the global MPN practice in Croatia; the proportion of physicians from academic centers was slightly higher than that in Italy (61.7%) [16]. We would like to point out, however, that specialized MPN hematology services are lacking in several community hospitals in Croatia. Therefore, many MPN patients from these geographic areas are transferred to physicians working in university hospitals. In this regard, and despite the fact that the majority of physicians included in this survey were from academic centers, the presented results may actually adequately capture the current MPN practice in our country.

Interestingly, two-thirds of Croatian hematologists also perform phlebotomies in *JAK2*-positive ET patients, as well as in primary MF and post-PV MF patients with hematocrit levels higher than 45%. This practice may not be entirely appropriate, due to the absence of formal recommendations from the current MPN treatment guidelines and the lack of confirmatory evidence from randomized clinical trials. Additionally, there is also the possibility of secondary iron-deficient anemia with subsequent platelet elevation in this patient population which may diminish patients’ quality of life and possibly lead to higher thrombotic risk. It is possible, however, that many physicians in Croatia perform phlebotomies for these patients due to the recently reported and underappreciated but non-negligible risk of thrombosis in this particular subset of MPN patients [9,21,22,23,24,25,26]. Patient-related factors (i.e., advanced atherosclerotic disease) and physician-perceived high thrombotic risk in this subset of MPN patients may also play a role. Nevertheless, prospective clinical trials are needed to unravel whether this practice may indeed improve outcomes in these patients.

Almost all hematologists in the survey responded they use direct oral anticoagulants in MPN patients, despite the fact that warfarin may still be the most frequently prescribed anticoagulant in MPNs globally. However, the results from this survey align with the accumulating pool of evidence regarding the excellent safety and efficacy of these compounds in this specific subset of patients [27,28,29,30,31]. Even though a very recent randomized trial (ARES) [32] has shown that twice-daily aspirin (100 mg *bis in die*-BID) may be superior to once-daily administration in ET patients, and the fact that renowned experts in the MPN field recommend the use of more intensive aspirin regimens in some ET and PV patients [1,2], twice- or thrice-daily aspirin is seldomly prescribed by Croatian hematologists. Some reasons may be the short follow-up period of the ARES trial (20 months) [32] precluding the conclusions regarding its safety and long-term effects on thrombotic risk, unavailability of pharmacodynamic assessments (i.e., serum thromboxane A_2_) in routine clinical practice, and the fact that the results from the ARES trial were extrapolated to PV patients without formal confirmatory evidence. Also, the final results of the ARES clinical trial have been published very recently, and it is plausible that they have not been fully embraced by the physicians in Croatia. It is also quite possible that further confirmatory evidence from other clinical trials, as well as more detailed real-life data on the safety and efficacy of these intensive aspirin regimens, is needed in order to reassure MPN physicians to change their long-term practice and fully incorporate these novel regimens into clinical practice. Notably, this is the first report from real-life MPN practice on how these intensive aspirin regimens are currently being embraced.

The survey also highlighted some areas of heterogeneity in daily practice in Croatia, especially in the treatment of MF-associated anemia and the frequency of cytogenetic and molecular testing. Most physicians treat MF-associated anemia with erythropoietin, followed by prednisone and immunomodulatory drugs (IMIDs, i.e., thalidomide or lenalidomide). The global use of erythropoietin for MF-associated anemia is frequent, particularly in patients with low serum erythropoietin levels. On the other hand, frequent use of prednisone and IMIDs for MF-associated anemia in Croatia may be caused by the lack of reimbursement of ruxolitinib for low- and intermediate-1-risk MF patients by Croatian Health Insurance, despite the fact that these patients may actually have disease-related anemia, symptomatic disease, or splenomegaly. Therefore, the use of these medications is most probably pursued in these lower risk MF patients or in patients with contraindications for ruxolitinib (i.e., concomitant solid tumors or infections). It is worth mentioning that similar erythropoietin (40%) and steroid (20.5%) utilization rates for MF-associated anemia were also noted in Italy [16].

Approximately half of surveyed physicians perform cytogenetic analyses and test for detrimental non-driver myeloid mutations in MF patients; however, even though the majority of physicians consider testing for non-driver myeloid mutations in MF and the *JAK2* allele burden in PV patients to be therapeutically and prognostically relevant, less than half of them actually order them in everyday practice. These results align with those from Italy, where approximately 30% of MF patients are routinely tested for molecular mutations at the time of disease diagnosis. The utilization of the MIPSS-70 prognostic score for risk stratification of MF patients, which incorporates molecular analyses, was also quite similar, and low (3.2% in Croatia and 4.9% in Italy) [16]. Moreover, only a minority of physicians caring for MPN patients repeat molecular analyses during follow-up. Similarly, almost all Croatian hematologists repeat bone marrow biopsies only at the time of disease progression suspicion, and not at regular time intervals. These observations are also quite similar to those from Italy, where approximately 50% of physicians caring for MPN patients order cytogenetic testing in cases of ET or PV suspicion, and where bone marrow biopsy is repeated only when disease progression is suspected (>90% of cases); the proportion of cytogenetic testing in MF was also quite similar (71.1% in Italy vs. 76.7% in Croatia) [16].

Considering the prospects of disease modification in MPNs (mostly assessed through achieving molecular remission and the normalization of bone marrow findings) [33,34,35], and the novel concepts regarding treatment-free remissions in MPN patients achieving long-term hematological and molecular responses [36], the imbalance in cytogenetic and molecular testing among academic and community centers probably indicates the lack of availability of specialized infrastructure for these assessments in the community setting. The inequality in MPN practice due to lack of availability of diagnostic resources and financial constraints may also pose a problem in many developing countries around the world. On the other hand, it could also reflect the uncertainties regarding the clinical relevance of repeated molecular analyses [37], especially outside clinical trials, that is, in real-life settings. Nevertheless, cytogenetic assessment may be prognostically relevant [1,2,3,4,14,15,38,39] and may be considered as a part of routine baseline MPN work-up. Therefore, the results of this survey highlight an important area where improvement in Croatian MPN practice is needed.

Finally, the results of this survey show that implementation of the MPN-SAF questionnaire in everyday practice is still far from being routinely used in daily practice in Croatia, despite its widespread use in randomized clinical trials; similar results have also been shown in a recent Italian survey [16]. The reasons for this observation may be MPN symptom underrecognizing and the discordance regarding treatment goals among physicians and patients [40,41].

A limitation of this study is its relatively low sample size; however, as stated before, Croatia is a small country and MPN patients from some geographic areas in Croatia do not have access to specialized MPN-related hematology care in their local community hospitals. In addition, many hematologists are not routinely involved in the management of MPN patients. Therefore, some MPN patients are transferred to physicians in university hospitals. For this reason, it is reasonable to conclude that the presented results may indeed provide an adequate snapshot of the current MPN practice in Croatia. Moreover, due to a smaller sample size, analyzing differences in clinical practice between academic and community centers may lack sufficient statistical power. Lastly, it should be pointed out that this survey primarily aimed to assess MPN daily practice, and not the respondents’ level of knowledge.

## 5. Conclusions

This study provides an important snapshot of the current MPN practice in Croatia and demonstrates a high level of abidance of Croatian physicians to international MPN diagnostic and treatment recommendations (see Supplementary Table S1). However, some areas of heterogeneity in MPN clinical practice have been noted, especially with respect to cytogenetic testing and enrollment of patients in clinical trials. Regular refreshments of national MPN guidelines, participation in international working groups on MPNs, patient engagement in MPN clinical trials and in MPN patient advocate groups, as well as organization and regular physician attendance of national and international meetings on MPNs in Croatia are needed to further improve MPN practice in Croatia. A parallel study by the KROHEM’s MPN Working Group Party regarding MPN epidemiology and MPN-related clinical outcomes in Croatia is also underway. Finally, due to the small sample size in the presented survey, similar studies from other countries are needed to provide a more detailed overview of real-life MPN practice globally.

## Data Availability

Data from this study are available upon reasonable request made to the corresponding author (Ivan Krecak).

## Supplementary Materials

The following supporting information can be downloaded at: https://www.mdpi.com/article/10.3390/jcm14051524/s1, Table S1. Full set of questions and answers from the online survey.

## References

[1] Tefferi A., Barbui T. (2023). Polycythemia vera: 2024 update on diagnosis, risk-stratification, and management. Am. J. Hematol..

[2] Tefferi A., Vannucchi A.M., Barbui T. (2024). Essential thrombocythemia: 2024 update on diagnosis, risk stratification, and management. Am. J. Hematol..

[3] Tefferi A. (2023). Primary myelofibrosis: 2023 update on diagnosis, risk-stratification, and management. Am. J. Hematol..

[4] Tefferi A., Rumi E., Finazzi G., Gisslinger H., Vannucchi A.M., Rodeghiero F., Randi M.L., Vaidya R., Cazzola M., Rambaldi A. (2013). Survival and prognosis among 1545 patients with contemporary polycythemia vera: An international study. Leukemia.

[5] Tefferi A., Guglielmelli P., Larson D.R., Finke C., Wassie E.A., Pieri L., Gangat N., Fjerza R., Belachew A.A., Lasho T.L. (2014). Long-term survival and blast transformation in molecularly annotated essential thrombocythemia, polycythemia vera, and myelofibrosis. Blood.

[6] Abu-Zeinah G., Silver R.T., Abu-Zeinah K., Scandura J.M. (2022). Normal life expectancy for polycythemia vera (PV) patients is possible. Leukemia.

[7] Barbui T., Tefferi A., Vannucchi A.M., Passamonti F., Silver R.T., Hoffman R., Verstovsek S., Mesa R., Kiladjian J.-J., Hehlmann R. (2018). Philadelphia chromosome-negative classical myeloproliferative neoplasms: Revised management recommendations from European LeukemiaNet. Leukemia.

[8] Marchetti M., Vannucchi A.M., Griesshammer M., Harrison C., Koschmieder S., Gisslinger H., Álvarez-Larrán A., De Stefano V., Guglielmelli P., Palandri F. (2022). Appropriate management of polycythaemia vera with cytoreductive drug therapy: European LeukemiaNet 2021 recommendations. Lancet Haematol..

[9] Haider M., Gangat N., Lasho T., Hussein A.K.A., Elala Y.C., Hanson C., Tefferi A. (2016). Validation of the revised International Prognostic Score of Thrombosis for Essential Thrombocythemia (IPSET-thrombosis) in 585 Mayo Clinic patients. Am. J. Hematol..

[10] Verstovsek S., Gotlib J., Mesa R.A., Vannucchi A.M., Kiladjian J.-J., Cervantes F., Harrison C.N., Paquette R., Sun W., Naim A. (2017). Long-term survival in patients treated with ruxolitinib for myelofibrosis: COMFORT-I and -II pooled analyses. J. Hematol. Oncol..

[11] Guglielmelli P., Ghirardi A., Carobbio A., Masciulli A., Maccari C., Mora B., Rumi E., Triguero A., Finazzi M.C., Pettersson H. (2022). Impact of ruxolitinib on survival of patients with myelofibrosis in the real world: Update of the ERNEST Study. Blood Adv..

[12] Masarova L., Bose P., Pemmaraju N., Daver N.G., Sasaki K., Chifotides H.T., Zhou L., Kantarjian H.M., Estrov Z., Verstovsek S. (2022). Improved survival of patients with myelofibrosis in the last decade: Single-center experience. Cancer.

[13] Tefferi A., Guglielmelli P., Lasho T.L., Coltro G., Finke C.M., Loscocco G.G., Sordi B., Szuber N., Rotunno G., Pacilli A. (2020). Mutation-enhanced international prognostic systems for essential thrombocythaemia and polycythaemia vera. Br. J. Haematol..

[14] Tefferi A., Guglielmelli P., Nicolosi M., Mannelli F., Mudireddy M., Bartalucci N., Finke C.M., Lasho T.L., Hanson C.A., Ketterling R.P. (2018). GIPSS: Genetically inspired prognostic scoring system for primary myelofibrosis. Leukemia.

[15] Tefferi A., Guglielmelli P., Lasho T.L., Gangat N., Ketterling R.P., Pardanani A., Vannucchi A.M. (2018). MIPSS70+ Version 2.0: Mutation and Karyotype-Enhanced International Prognostic Scoring System for Primary Myelofibrosis. J. Clin. Oncol..

[16] Loscocco G.G., Mannelli F., Guglielmelli P., Paoli C., Marone I., Cucci R., Coltro G., Sordi B., Albano F., Breccia M. (2019). A GIMEMA Myeloproliferative Neoplasms Working Party initiative. Am. J. Hematol..

[17] Jentsch-Ullrich K., Eberhardt J., Zeremski V., Koehler M., Wolleschak D., Heidel F.H. (2016). Characteristics and treatment of polycythemia vera patients in clinical practice: A multicenter chart review on 1476 individuals in Germany. J. Cancer Res. Clin. Oncol..

[18] Habib L.A., Kuo K.H.M., Panzarella T., Gupta V., Trinkaus M. (2019). Management of Polycythemia Vera: A Survey of Canadian Physician Practice Patterns. Clin. Lymphoma Myeloma Leuk..

[19] Kander E.M., Moliterno A.R., Rademaker A., Streiff M.B., Spivak J.L., Stein B.L. (2016). Practice Patterns in the Diagnosis and Treatment of Polycythemia Vera in the Post-JAK2 V617F Discovery Era. J. Natl. Compr. Canc Netw..

[20] Arber D.A., Orazi A., Hasserjian R., Thiele J., Borowitz M.J., Le Beau M.M., Bloomfield C.D., Cazzola M., Vardiman J.W. (2016). The 2016 revision to the World Health Organization classification of myeloid neoplasms and acute leukemia. Blood.

[21] Barbui T., Carobbio A., Cervantes F., Vannucchi A.M., Guglielmelli P., Antonioli E., Alvarez-Larrán A., Rambaldi A., Finazzi G., Barosi G. (2010). Thrombosis in primary myelofibrosis: Incidence and risk factors. Blood.

[22] Kc D., Falchi L., Verstovsek S. (2017). The underappreciated risk of thrombosis and bleeding in patients with myelofibrosis: A review. Ann. Hematol..

[23] Mora B., Guglielmelli P., Kuykendall A., Rumi E., Maffioli M., Palandri F., De Stefano V., Caramella M., Salmoiraghi S., Kiladjian J.-J. (2022). Prediction of thrombosis in post-polycythemia vera and post-essential thrombocythemia myelofibrosis: A study on 1258 patients. Leukemia.

[24] Hernández-Boluda J., Pastor-Galán I., Arellano-Rodrigo E., Raya J., Pérez-Encinas M., Ayala R., Ferrer-Marín F., Velez P., Mora E., Fox M. (2022). Predictors of thrombosis and bleeding in 1613 myelofibrosis patients from the Spanish Registry of Myelofibrosis. Br. J. Haematol..

[25] Lucijanic M., Krecak I., Soric E., Sabljic A., Galusic D., Holik H., Perisa V., Peric M.M., Zekanovic I., Kusec R. (2022). Patients with post polycythemia vera myelofibrosis might experience increased thrombotic risk in comparison to primary and post essential thrombocythemia myelofibrosis. Leuk. Res..

[26] Barbui T., Ghirardi A., Carobbio A., Masciulli A., Carioli G., Rambaldi A., Finazzi M.C., Bellini M., Rumi E., Vanni D. (2022). Increased risk of thrombosis in JAK2 V617F-positive patients with primary myelofibrosis and interaction of the mutation with the IPSS score. Blood Cancer J..

[27] Barbui T., De Stefano V., Carobbio A., Iurlo A., Alvarez-Larran A., Cuevas B., Marín F.F., Vannucchi A.M., Palandri F., Harrison C. (2021). Direct oral anticoagulants for myeloproliferative neoplasms: Results from an international study on 442 patients. Leukemia.

[28] How J., Story C., Ren S., Neuberg D., Rosovsky R.P., Hobbs G.S., Connors J.M. (2021). Practice patterns and outcomes of direct oral anticoagulant use in myeloproliferative neoplasm patients. Blood Cancer J..

[29] Baysal M., Aksoy E., Bedir K.H., Özmen D., Patır P., Demirci U., Yaman S., Özdemir Z.N., Gürsoy V., Yıldızhan E. (2024). Real-world data on direct oral anticoagulants in BCR::ABL1-negative myeloproliferative neoplasms (MPNs): A multicenter retrospective study on behalf of scientific subcommittee on MPNs for Turkish society of hematology. J. Thromb. Thrombolysis..

[30] Lee J.Y., Lee J.H., Park W., Seo J., Kang M., Jung E.H., Kim S.-A., Suh K.J., Kim J.-W., Kim S.H. (2024). The Role of Direct Oral Anticoagulants in Managing Myeloproliferative Neoplasms Patients. Cancer Res. Treat..

[31] Baysal M., Bayrak M., Eşkazan A.E. (2023). Current evidence on the use of direct oral anticoagulants in patients with myeloproliferative neoplasm: A systematic review. Expert Rev. Hematol..

[32] Rocca B., Tosetto A., Petrucci G., Rossi E., Betti S., Soldati D., Iurlo A., Cattaneo D., Bucelli C., Dragani A. (2024). Long-term pharmacodynamic and clinical effects of twice- versus once-daily low-dose aspirin in essential thrombocythemia: The ARES trial. Am. J. Hematol..

[33] Harrison C.N. (2024). Are we ready for disease modification in myeloproliferative neoplasms?. Hemasphere.

[34] Vachhani P., Verstovsek S., Bose P. (2022). Disease Modification in Myelofibrosis: An Elusive Goal?. J. Clin. Oncol..

[35] Vannucchi A.M., Pieri L., Guglielmelli P. (2011). JAK2 Allele Burden in the Myeloproliferative Neoplasms: Effects on Phenotype, Prognosis and Change with Treatment. Ther. Adv. Hematol..

[36] De Oliviera R.D., Soret-Dulphy J., Zhao L.-P., Marcault C., Gauthier N., Verger E., Maslah N., Parquet N., Raffoux E., Vainchenker W. (2021). Interferon-alpha (IFN) therapy discontinuation is feasible in myeloproliferative neoplasm (MPN) patients with complete hematological remission [abstract]. Blood.

[37] Tefferi A., Pardanani A., Gangat N. (2024). Treatment-associated decline in JAK2V617F allele burden in polycythemia vera: What does it mean?. Am. J. Hematol..

[38] Gangat N., Jadoon Y., Szuber N., Hanson C.A., Wolanskyj-Spinner A.P., Ketterling R.P., Pardanani A., Tefferi A. (2022). Cytogenetic abnormalities in essential thrombocythemia: Clinical and molecular correlates and prognostic relevance in 809 informative cases. Blood Cancer J..

[39] Barraco D., Cerquozzi S., Hanson C.A., Ketterling R.P., Pardanani A.D., Gangat N., Tefferi A. (2018). Cytogenetic findings in WHO-defined polycythaemia vera and their prognostic relevance. Br. J. Haematol..

[40] Scherber R., Dueck A.C., Johansson P., Barbui T., Barosi G., Vannucchi A.M., Passamonti F., Andreasson B., Ferarri M.L., Rambaldi A. (2011). The Myeloproliferative Neoplasm Symptom Assessment Form (MPN-SAF): International prospective validation and reliability trial in 402 patients. Blood.

[41] Mesa R.A., Miller C.B., Thyne M., Mangan J., Goldberger S., Fazal S., Ma X., Wilson W., Paranagama D.C., Dubinski D.G. (2017). Differences in treatment goals and perception of symptom burden between patients with myeloproliferative neoplasms (MPNs) and hematologists/oncologists in the United States: Findings from the MPN Landmark survey. Cancer.

