# Supplementary material for: Survey of Clinical Practice in Chronic Myeloproliferative Neoplasms in Croatia: A Study by the MPN Working Group Party of the Croatian Cooperative Group for Hematologic Diseases (KROHEM)
Source: J Clin Med. 2025 Feb 24;14(5):1524. doi: 10.3390/jcm14051524 (PMC11900499; doi:10.3390/jcm14051524)
Supplement: Supplementary file 1 [file jcm-14-01524-s001.zip › jcm-3438939-supplementary.pdf]

Domain 1: Physician characteristics (total number =31)	
**1. Sex**	
a) Female	13 (41.9%)
b) Male	18 (58.1%)
**2. Where do you practice your clinical activity?**	
a) University hospital	22 (71%)
b) General hospital	9 (29%)
c) Private practice	0%
**3. How many years of experience do you have in the field of MPNs? (n=30)**	
a) <1 year	0%
b) 1-5 years	2 (6.7%)
c) 6-9 years	12 (40%)
d) >10 years	16 (53.3%)
**4. How many PV patients do you follow in your clinical practice? (n=30)**	
a) 1-10	9 (30%)
b) 11-20	11 (36.7%)
c) 21-30	4 (13.3%)
d) >30	6 (20%)
**5. How many ET patients do you follow in your clinical practice?**	
a) 1-10	13 (41.9%)
b) 11-20	6 (19.4%)
c) 21-30	6 (19.4%)
d) >30	6 (19.4%)
**6. How many MF patients do you follow in your clinical practice?**	
a) 1-5	19 (61.3%)
b) 6-10	8 (25.8%)
c) 11-20	2 (6.5%)
d) >20	2 (6.5%)
**7. How many post-MPN acute leukemia patients do you see per year in your clinical practice?**	
a) 0	11 (35.5%)
b) 1-5	16 (51.6%)
c) 6-10	4 (12.9%)
d) >10	0%
**8. How many MF patients do you transfer to allotransplant per year?**	
a) 0	14 (45.2%)
b) 1-5	16 (51.6%)
c) 6-10	1 (3.2%)
d) >10	0%
**Domain 2: Diagnostic procedures (total number=31)**	
**9. Do you routinely screen for BCR::ABL1 mutation in MPN suspicion?**	
**a**) Yes	21 (67.7%)
b) No	10 (32.3%)

**10. What tests do you perform in PV suspicion?***	
a) JAK2-V617F	31 (100%)
b) Serum erythropoietin	26 (83.9%)
c) Bone marrow biopsy	24 (77.4%)
d) Cytogenetics	16 (51.6%)
**11. In case of PV suspicion, do you test all patients for the presence of exon12 mutation in case V617F mutation is negative?**	
a) Yes	17 (54.8%)
b) No	14 (45.2%)
**12. In your opinion, JAK2-V617F allele burden measurement is important for? * (n=30)**	
a) Correct MPN diagnosis	16 (53.3%)
b) Assessment of treatment	16 (53.3%)
c) Disease prognosis	17 (56.7%)
d) Not relevant	6 (20%)
**13. Do you routinely test for CALR and MPL mutations in ET and MF patients if JAK2-V617F mutation is negative?**	
a) Yes	29 (93.5%)
b) No	2 (6.5%)
**14. Do you routinely perform bone marrow biopsy in ET suspicion?**	
a) Yes	23 (74.2%)
b) No	8 (25.8%)
**15. Do you routinely perform cytogenetics in ET and PV patients?**	
a) Yes	17 (54.8%)
b) No	14 (45.2%)
**16. Do you routinely perform cytogenetics in MF patients? (n=30)**	23 (76.7%)
a) Yes	7 (23.3%)
b) No	
**17. Do you routinely test for non-driver mutations in MF patients?**	
a) Yes	14 (45.2%)
b) No	17 (54.8%)
**18. Do you routinely assess thrombotic risk stratification in PV patients (two variables: age> 60 and/or prior thrombosis)?**	26 (83.8%)
a) Yes	5 (16.1%)
b) No	
**19. Do you routinely assess thrombotic risk stratification in ET patients according to R-IPSET score? (three variables: age> 60, prior thrombosis, JAK2-V617F mutation)**	
a) Yes	26 (83.9%)
b) No	5 (16.1%)
**20. For risk stratification of MF patients which risk score do you prefer to use?**	
a) IPSS	7 (22.6%)
b) DIPSS	20 (64.5%)

c) DIPSS-Plus<br>d) MIPSS	3 (9.7%)<br>1 (3.2%)
**21. Do you consider important testing for detrimental myeloid mutations such as ASXL1, EZH2, SRSF2 and IDH1/IDH2, to assess the risk in younger patients with MF? (n=30)**<br>a) Yes<br>b) No	 <br><br>26 (86.7%)<br>4 (13.3%)
**Domain 3: Therapeutic decisions (total number=31)**	
**22. What is target haematocrit in your practice for phlebotomy in PV patients?**<br>a) <42% for woman and <45% for men<br>b) <45% (no difference by gender)<br>c) <50%<br>d) 50-55%	 <br>7 (22.6%)<br>22 (71%)<br>2 (6.5%)<br>0%
**23. Do you perform phlebotomies in JAK2-positive ET patients with hematocrit >45%?**<br>a) Yes<br>b) No	 <br>21 (67.7%)<br>10 (32.3%)
**24. Do you perform phlebotomies in JAK2-positive primary MF or post-PV MF patients with hematocrit >45%?**<br>a) Yes<br>b) No	 <br>21 (67.7%)<br>10 (32.3%)
**25. Do you recommed aspirin to all PV patients?**<br>a) Yes<br>b) No	 29 (93.5%)<br>2 (6.5%)
**26. Do you recommed aspirin to all ET patients?**<br>a) Yes<br>b) No	 22 (71%)<br>9 (29%)
**27. To which ET patients you do NOT recommend aspirin?* (n=26)**<br>a) < 40 years of age<br>b) < 60 years, JAK2-unmutated, no prior thrombosis<br>c) CALR-mutated	 <br>13 (50%)<br>18 (69.2%)<br>6 (23.1)
**28. To which MF patients do you recommend aspirin?***<br>a) With thrombosis history<br>b) JAK2-V617F mutated<br>c) With Post-PV MF<br>d) With concomitant cardiovascular risk factors	 21 (67.7%)<br>18 (61.3%)<br>14 (48.4%)<br>28 (90.3%)
**29. Do you recommend twice- or thrice-daily aspirin to some ET patients?**<br>a) Yes<br>b) No	 <br>2 (6.5%)<br>29 (93.5%)
**30. Except for high-risk ET and PV patients, what are other conditions for which you recommend cytoreduction?***<br>a) Leukocytosis<br>b) Concomitant cardiovascular risk factors<br>c) Splenomegaly<br>d) Disease-related symptoms	 <br>25 (80.6%)<br>12 (38.7%)<br>13 (41.9%)<br>19 (61.3%)
**31. Which is the first line agent you use for cytoreduction in PV patients?**	

a) Hydroxyurea<br>b) Interferons<br>c) Ruxolitinib<br>d) Other	27 (87.1%)<br>4 (12.9%)<br>0%<br>0%
**32. Which is the second line agent you use for cytoreduction in PV patients?**<br>a) Hydroxyurea<br>b) Interferons<br>c) Busulfan<br>d) Ruxolitinib	 <br>2 (6.5%)<br>19 (61.3%)<br>10 (32.3%)<br>0%
**33. Which is the first line agent you use for cytoreduction in ET patients? (n=30)**<br>a) Hydroxyurea<br>b) Interferons<br>c) Anagrelide<br>d) Other	 <br>24 (80%)<br>4 (13.3%)<br>2 (6.7%)<br>0%)
**34. Which is the second line agent you use for cytoreduction in ET patients?**<br>a) Hydroxyurea<br>b) Interferons<br>c) Anagrelide<br>d) Busulfan<br>e) Ruxolitinib	 <br>5 (16.1%)<br>13 (48.4%)<br>7 (25.8%)<br>0%<br>3 (9.7%)
**35. Which one of the following agents do you usually use to treat MF-associated anemia?**<br>a) Erythropoietin<br>b) Thalidomide/lenalidomide<br>c) Prednisone<br>d) Danazol	 <br>16 (51.6%)<br>6 (19.4%)<br>7 (22.6%)<br>2 (6.5%)
**36. Which one of the following agents do you usually use to manage splenomegaly in MF?**<br>a) Ruxolitinib<br>b) Hydroxyurea<br>c) Interferons<br>d) Other	 <br>29 (93.5%)<br>2 (6.5%)<br>0%<br>0%
**37. Which is the indication for splenectomy in MF patients in your clinical practice?***<br>a) Painful splenomegaly refractory to cytoreduction and irradiation<br>b) Portal hypertension<br>c) Splenic infarction<br>d) Severe refractory anemia and/or thrombocytopenia	 <br>30 (96.8%)<br><br>7 (29%)<br>9 (29%)<br>13 (41.9%)
**38. Which is the most common induction treatment for post-MPN acute leukemia patients in your clinical practice?* (n=30)**<br>a) 3+7<br>b) Azacitidine and venetoclax<br>c) High dose cytarabine<br>d) Palliation<br>e) Other	 <br><br>13 (43.3%)<br>22 (73.3%)<br>0%<br>7 (23.3%)<br>7 (23.3%)

**39. Do you use direct oral anticoagulants (non coumadin) in MPN patients?**<br>a) Yes<br>b) No	 <br>29 (93.5%)<br>2 (6.5%)
**40. Have you enrolled MPN patients in clinical trials at your center?**<br>a) Yes<br>b) No	 <br>21(67.7%)<br>10 (32.3%)
**Domain 4: Follow-up (total number=31)**	
**41. At what intervals do you usually visit low-risk PV patients in regular follow up?**<br>a) Monthly<br>b) Every 3 months<br>c) Every 6 months<br>d) Every 12 months	 <br>1 (3.2%)<br>23 (74.2%)<br>7 (22.6%)<br>0%
**42. At what intervals do you usually visit high-risk PV patients in regular follow up?**<br>a) Monthly<br>b) Every 3 months<br>c) Every 6 months<br>d) Ever 12 months	 <br>15 (48.4%)<br>16 (51.6%)<br>0%<br>0%
**43. At what intervals do you usually visit low-risk ET patients in regular follow up?**<br>a) Monthly<br>b) Every 3 months<br>c) Every 6 months<br>d) Every 12 months	 <br>0%<br>18 (58.1%)<br>12 (38.7%)<br>1 (3.2%)
**44. At what intervals do you usually visit high-risk ET patients in regular follow up?**<br>a) Monthly<br>b) Every 3 months<br>c) Every 6 months<br>d) Every 12 months	 <br>10 (32.3%)<br>21 (67.7%)<br>0%<br>0%
**45. At what intervals do you usually visit low/intermediate-1 (according to IPSS and DIPSS) risk MF patients in regular follow up?**<br>a) Monthly<br>b) Every 3 months<br>c) Every 6 months<br>d) Every 12 months	 <br><br>4 (12.9%)<br>21 (67.7%)<br>6 (19.4)<br>0%
**46. At what intervals do you usually visit intermediate-2/high-risk (according to IPSS and DIPSS) risk MF patients in regular follow up?**<br>a) Monthly<br>b) Every 3 months<br>c) Every 6 months<br>d) Every 12 months	 <br><br>21 (67.7%)<br>10 (32.3%)<br>0%<br>0%
**47. How often do you repeat bone marrow biopsies?**<br>a) Every 12 months<br>b) Every 3-5 years	 0%<br>3 (9.7%)

c) At suspicion of disease transformation<br>d) Never	28 (90.3%)<br>0%
**48. Do you repeat myeloid mutations testing during follow-up?**<br>a) Yes<br>b) No	 <br>4 (12.9%)<br>27 (87.1%)
**49. Which methods do your prefer for spleen size assessments?**<br>a) Palpation<br>b) Ultrasound<br>c) Computerized tomography<br>d) Magnetic resonance	 8 (25.8%)<br>16 (51.6%)<br>7 (22.6%)<br>0%
**50. Do you use MPN-SAF questionnaire in your clinical routine work?**<br>a) Yes<br>b) No	 <br>5 (16.1%)<br>26 (83.9%)
*Multiple answers possible. MPNs=myeloproliferative neoplasms, PV=polycythemia vera, ET=essential thrombocythemia, MF=myelofibrosis, R-IPSET=Revised International Prognostic Score of Thrombosis in Essential Thrombocythemia, IPSS=International Prognostic Scoring System, DIPSS=Dynamic International Prognostic Systemic Scoring MIPSS=Mutation Enhanced International Prognostic Systemic Scoring MPN-SAF=Myeloproliferative Neoplasm Symptom Assessment Form.	
